# Evolution of starvation resistance in an invasive insect species, *Aethina tumida* (Coleoptera: Nitidulidae)

**DOI:** 10.1002/ece3.6605

**Published:** 2020-07-23

**Authors:** Anna Papach, Geoffrey R. Williams, Peter Neumann

**Affiliations:** ^1^ Institute of Bee Health, Vetsuisse Faculty University of Bern Bern Switzerland; ^2^ Department of Entomology & Plant Pathology Auburn University Auburn AL USA; ^3^ Swiss Bee Research Centre Agroscope Bern Switzerland

**Keywords:** adaptation, invasive, small hive beetle, starvation resistance

## Abstract

Starvation resistance, or the ability to survive periods without food, can shed light on selection pressure imposed by food scarcity, including chances to invade new regions as a result of human transport. Surprisingly, little information is known about starvation resistance for invasive insect species. Given that native and invasive populations differ in starvation resistance, this would suggest different selection scenarios and adaptive shifts fostering invasion success. Here, we show striking differences in starvation resistance of adult small hive beetles *Aethina tumida* (SHB) between native and invasive populations. In the laboratory, starvation resistance of freshly emerged laboratory‐reared and field‐collected adult females and males was evaluated in the beetle's native African range and in their invasive North American range. SHB in their native African range survived longer than SHB in their invasive North American range. Across ranges, females survived longer than males. Field‐collected SHB survived in Africa longer than freshly emerged ones, but not in the invasive range. This suggests no selection for starvation resistance in the invasive range, possibly due to differences between African and European‐derived honey bee hosts facilitating a trade‐off scenario between reproduction and starvation resistance. The ability of adult females to survive up to two months without food appears to be one factor contributing to the invasion success of this species. Assuming food availability is usually high in the invasive ranges, and trade‐offs between starvation resistance and fecundity/reproduction are common, it seems as if selection for starvation resistance during transport could set up potential trade‐offs that enhance reproduction after invasion. It would be interesting to see if this is a possible general pattern for invasive insect species.

## Graphical abstract

The ability to survive periods without food can shed light on selection pressure imposed by food scarcity, including changed selection scenarios for invasive species in native and novel ranges. Field‐collected and freshly emerged adult small hive beetles *Aethina tumida* (SHB) survived longer in their native African range compared to the invasive range in North America. The data suggest no selection for starvation resistance in the invasive range, possibly contributing to invasion success of this species.

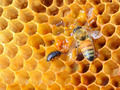

## 1 INTRODUCTION

Most animals experience some periods of food scarcity during their lifetime. To survive such unfavorable environmental conditions, animals may enter into diapause (Horton & Capinera, 1988), hibernate (Watt, Orttsland, Jonkel, & Ronald, 1981), or evolve other traits to increase their starvation resistance (Hoffmann & Harshman, 1999; Rion & Kawecki, 2007). Starvation resistance is a phenotypic trait that is usually measured as the time an animal can survive under complete food deprivation (Hoffmann & Harshman, 1999). It reflects the environment an animal lives in and its life history, and can vary tremendously from less than a day (adult mayflies Cranshaw & Redak, 2013) to more than a year (lone star ticks, Jaworski, Sauer, Williams, McNew, & Hair, 1984). Within the same species, there can also be differences in how an individual cope with starvation, including age, size, gender, and previous nutrition status (Rion & Kawecki, 2007). For example, female insects tend to have superior starvation resistance compared to males (Aggarwal, 2014; Lehmann et al., 2006; Reim, Teuschl, & Blanckenhorn, 2006; Renault, Hance, Vannier, & Vernon, 2003).

Invasive species provide a good opportunity to study the evolution of specific fitness‐related traits (Colautti & Barrett, 2013). Very often species that establish in new places exhibit high phenotypic plasticity and undergo rapid evolution that improves their survival and success in a novel environment (Sexton, McKay, & Sala, 2002; Whitney & Gabler, 2008). Due to new biotic and abiotic environments, invasive species often evolve novel traits yet reduce others that become less relevant for fitness (Colautti & Lau, 2015). For example, white champion plants invested in more flowers and faster germination, and less in defense traits, when they were introduced to North America from Europe (Blair & Wolfe, 2004). One phenotypical trait that might contribute to the success of invasive species is starvation resistance, as this enhances chances to spread (Laparie, Larvor, Frenot, & Renault, 2012; Moreira & Spata, 2002; Wu, Li, & Liu, 2016). Animals with higher starvation resistance are probably much more likely to reach new destinations via human‐assisted long‐range transport and survive from several days to several months under food deprivation until a new food source will eventually be available.

Small hive beetle (SHB), *Aethina tumida*, is an invasive species originating from sub‐Saharan Africa (Lundie, 1940) that has spread almost globally (Neumann, Pettis, & Schäfer, 2016; Ouessou Idrissou, Huang, Yañez, & Neumann, 2019). The SHB lives in association with honey bee colonies, but can also infest colonies of other social bees as well as solitary bee nests (Gonthier et al., 2019; Neumann et al., 2016). Inside host nests, SHB adults can feed themselves on honey, pollen, host brood, dead or live adult bees or tricking honey bee workers into trophallactic feeding, mate and reproduce (Neumann et al., 2016). Outside host nests, adult SHB can exploit a wide range of alternative food sources, including foraging on flowers (Buchholz et al., 2008; Gonthier et al., 2019). An adult mated female SHB can lay up to 2000 eggs in her lifetime (Arbogast, Torto, & Teal, 2010; de Guzman, Rinderer, & Frake, 2015). Once an egg hatches, the SHB larvae will feed on any suitable food available (honey, pollen, bee brood, fruits and even rotten schnitzel (i.e., decomposing meat; Buchholz et al., 2008; Neumann et al., 2016) until it reaches the post‐feeding larval stage (wandering stage, Lundie, 1940). It will then pupate in suitable nearby soil, thereby completing the life cycle (Neumann & Elzen, 2004). An adult emerging from the soil can conduct a long‐range flight to reach a new host colony (Neumann, Hoffmann, Duncan, Spooner‐Hart, & Pettis, 2012). Since the adult emerging from the soil will have just completed pupation, a limited amount of energy reserves is expected to remain (Llandres et al., 2015).

Within its native range, SHB is usually considered to be a minor pest (Hepburn & Radloff, 1998), while in the invasive ranges, it can cause considerable damage, often leading to honey bee colony collapse (Spiewok, Duncan, Spooner‐Hart, Pettis, & Neumann, 2008). Quantitative differences in a range of defense behaviors between African and European (‐derived) honey bee hosts appear to underlie the differential pest impact of SHB (Neumann & Elzen, 2004). For example, African honey bee workers more readily attack SHB (Elzen et al., 2001), thereby limiting their movement in colonies. Moreover, honey bee workers incarcerate adult SHB in propolis prisons (Neumann et al., 2001). Colonies of European honey bee subspecies are less efficient in preparing for SHB‐induced swarming (absconding) compared to African ones and are leaving ample protein food behind (Neumann et al., 2018). These results in striking differences by two orders of magnitude in SHB reproduction between abandoned nests of African and European honey bee subspecies (Neumann et al., 2018).

Here, we compared starvation resistance of SHBs from a population in the native range (Republic of South Africa = RSA) with an invasive one (USA). In our experimental design, we decided to compare field‐collected versus freshly emerged, laboratory‐reared SHB to better understand the ability of adults to cope with starvation. The rationale behind comparing field‐collected versus freshly emerged, laboratory‐reared SHB was that prior to collection, field‐collected adults will almost certainly have consumed food after emergence, while freshly emerged laboratory ones could have not. Since phenotype is usually influenced by genotype and environment, the comparison between freshly emerged and field‐collected adults under identical environmental conditions in the laboratory enabled us to estimate the impact of genetics (freshly emerged) versus environment (i.e., field‐caught from African versus European‐derived honey bee host colonies) for starvation resistance. First, we predicted that SHB in the novel range will have a higher starvation resistance than in the native range since founder SHB had to survive a period of transportation and food absence; therefore, only SHB with high starvation resistance would be selected. Second, considering that novel environment differs and changes selection scenarios, we expected to see differences between field‐caught adult SHBs from the native and invasive range. Moreover, field‐caught adults are expected to have a superior starvation resistance compared to freshly emerged ones given that there is selection for starvation resistance.

## 2 MATERIALS AND METHODS

The experiments were conducted at Auburn University, AL, USA and at Rhodes University, Grahamstown, Republic of South Africa (RSA). Experimental adult SHB were manually collected (Neumann et al., 2013) during local summer from naturally infested local field colonies of mixed European origin (predominantly *A. mellifera ligustica*, USA) in 2019 or of African subspecies (Cape honey bee *A. m. capensis *x *A. m. scutellata* hybrids, RSA) in 2001. All experiments at both locations were performed following the exact same protocols as described below.

### 2.1 Starvation of laboratory‐reared beetles

Field‐collected SHB (seven females and seven males) were used to establish a laboratory rearing following standard protocols (Neumann et al., 2013). The first generation of laboratory‐reared adults was then used for the experiments. Upon emergence, adult beetles were sexed (Schmolke, 1974) and individually placed in standard Eppendorf reaction tubes [1.5 ml, *N* = 48‐50/sex/location] with punctured lids to avoid suffocation. Tubes with beetles were kept at 25°C and 80% relative humidity (RH) in an incubator; every other day, they were provided with a drop of water to limit dehydration. Adult SHB mortality was recorded daily until all experimental individuals have died.

### 2.2 Starvation of field‐collected beetles

Adult SHB were collected from naturally infested local honey bee colonies (see above). As SHB were randomly collected from the field, their age and previous nutrition status were unknown. All collected beetles were sexed (Schmolke, 1974), placed in 1.5 ml Eppendorf reaction tubes [*N* = 49‐50/sex/location], and kept as described above. Tubes were inspected daily and a number of dead individuals were recorded until the last beetles have died.

### 2.3 Statistical analyses

Survival times of male and female SHBs for both locations and both groups (laboratory‐reared versus field‐collected) were fitted using the mestreg function for multilevel survival models (StataCorp, 2017). “Location,” “Origin,” and “Sex” were included as fixed variables. Median longevity was calculated as the 50th percentile of the survival time. Survival analyses and all calculations were performed using STATA 15. All statistical figures were created using the R version 3.5.1 (R Core Team).

## 3 RESULTS

**FIGURE 1:**
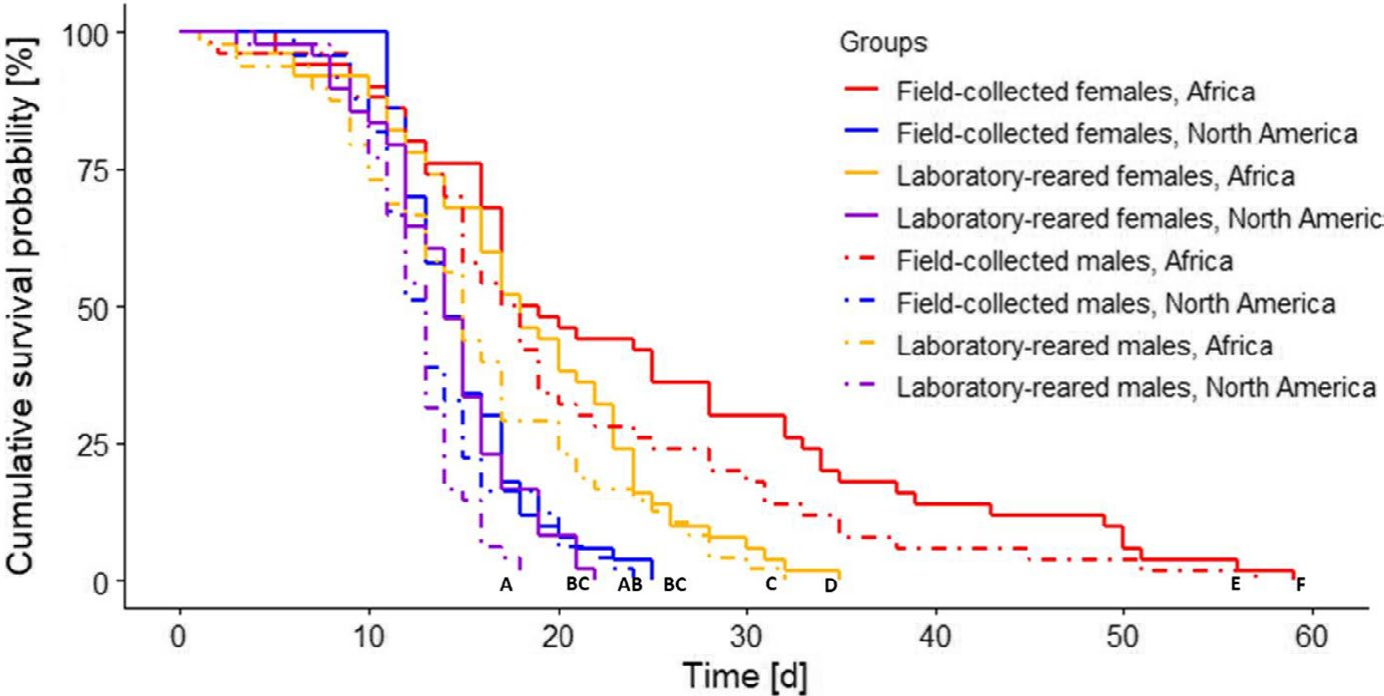
Mortality of laboratory‐reared and field‐collected adult small hive beetles, *Aethina tumida*, under starvation from the endemic range in Africa and from the invasive range in North America. Kaplan–Meier survival curves show the cumulative survival (%) in days [d] of all groups (Africa: laboratory‐reared females and males, field‐collected females and males; North America: laboratory‐reared females and males, field‐collected females and males). Significant differences between groups (*p < *.05) are indicated by different capital letters. Curves sharing letters are not significantly different from each other

Starvation resistance of adult SHBs was significantly influenced by location, origin, and gender. In general, SHB from the native range outlived beetles from the invasive range; field‐collected beetles survived longer than laboratory‐reared ones, and females had better survival than males (Figure 1).

Median longevity of field‐collected females in the native range (Table 1) was significantly higher compared to all other groups: field‐collected males in the native range (*p* = .01), laboratory‐reared females in the native range (*p* < .001), laboratory‐reared males in the native range (*p* < .01), field‐collected females in the invasive range (*p* < .001), field‐collected males in the invasive range (*p* < .001), laboratory‐reared females in the invasive range (*p* < .001), and laboratory‐reared males in the invasive range (*p* < .001). Laboratory‐reared males in the invasive range had the lowest starvation resistance with beetles surviving up to day 18, and the highest starvation resistance was observed in field‐collected females in the native range with individuals surviving up to day 59 (Figure 1).

**TABLE 1:** Mortality of laboratory‐reared and field‐collected adult small hive beetles, *Aethina tumida,* under starvation from the endemic range in Africa and from the invasive range in North America

Groups	*N*	Median longevity (days)	95% confidence interval	
			Lower	Upper
Field‐collected females, Africa	50	18	17	28
Field‐collected males, Africa	50	17	15	20
Laboratory‐reared females, Africa	50	18	16	22
Laboratory‐reared males, Africa	48	15	13	17
Field‐collected females, North America	50	14	13	16
Field‐collected males, North America	49	13	12	14
Laboratory‐reared females, North America	48	14	13	15
Laboratory‐reared males, North America	48	13	12	13

Note

The experimental groups, sample sizes [*N*], median longevity [days], and 95% confidence intervals are shown.

In the native range, field‐collected SHBs survived significantly longer than the ones reared in the laboratory (*p* < .001). Interestingly, in the invasive range, starvation resistance of laboratory‐reared beetles did not significantly differ from the starvation resistance of field‐collected ones (*p* = .121). In the invasive range, there was no difference in starvation resistance between both laboratory‐reared and field‐collected females and males (for both *p* = 1). Moreover, field‐collected females in the invasive range had similar survival probability compared to laboratory‐reared males in the native range (*p* = 1). Starvation resistance of laboratory‐reared females in the invasive range did not differ from the starvation resistance of field‐collected males in the invasive range (*p* = 1) and from laboratory‐reared males in the native range (*p* = .102).

## 4 DISCUSSION

The data show striking differences in starvation resistance between SHB from the native range in Africa and the invasive range in North America. In sharp contrast to our expectations, SHB in their native African range survived longer than SHB in their invasive North American range. Moreover, field‐collected SHB survived in Africa longer than freshly emerged ones, but not in the invasive North American range. Even freshly emerged African SHB survived longer than field‐collected ones in North America. Across ranges, female SHB also tend to survive longer than males independent of location, possibly reflecting differences in body size (Ellis, Delaplane, Hood, 2002).

It is obvious that differences in survival could not only be explained by starvation resistance, but also due to genetically determined longevity caused by any other mechanism (e.g., antioxidant gene expression; Arking et al., 2000). However, the standard laboratory conditions (Neumann et al., 2013) were identical for all treatments and suitable for adult maintenance of SHB given that food would also have been provided. It therefore appears as if lack of food was the major stress factor; therefore, starvation resistance was the key mechanism governing survival of the experimental adult SHB. In principle, an experimental treatment without starvation might have worked as a control treatment. This was not feasible though as adult female SHB provided with adequate food (Neumann et al., 2013), but not allowed to oviposit (see Neumann et al., 2016), can live for more than one year in the laboratory (data not shown). The latter observation also suggests that mechanisms other than starvation resistance (e.g., senescence) are of minor importance in this particular laboratory context.

It must also be pointed out that the present study is only based on a comparison of two populations, one from the native and one from the invasive range. Therefore, the results have to be interpreted with caution and should not be generalized for the entire native and invasive ranges of SHB. Indeed, it seems likely that there are differences in starvation resistance between endemic as well as invasive populations due to the vast distribution range of SHB in sub‐Saharan Africa ranging from deserts to rain forests (see below, Neumann et al., 2016). Moreover, genetic bottlenecks, genetic drift, and inbreeding are likely to contribute due to the usual small founding size of invasive populations (Hamilton, 2009). Nevertheless, these first results comparing starvation resistance of invasive and native SHB populations indicate possible intriguing differences, which may foster invasion success.

Previous studies mainly focused on survival of laboratory‐reared adult SHB maintained with water as negative controls (Schmolke, 1974 (RSA); Ellis, Neumann, et al., 2002 (RSA); Buchholz et al., 2008 (Maryland, USA); Gonthier et al., 2019 (Alabama, USA). In those experiments, SHBs maintained with water only survived for up to 14 days (Ellis, Neumann, et al., 2002), 19 days (Schmolke, 1974), 19 days (Gonthier et al., 2019), and 26 days (Buchholz et al., 2008); this overall corresponds with the results reported here. However, these previous studies used varying methods and did neither compare freshly emerged laboratory‐reared versus field‐caught adults, males versus females nor endemic versus invasive ranges of SHB. There is a temporal difference in data acquisition (RSA: 2001, USA: 2019). However, we believe that those temporal differences should not result in notable changes as selection scenarios within the native range SHB did not change (like new hosts in North America or host density). Therefore, the data reflect a general trend for SHB in a given region.

The data show for the first time that female SHB have a higher longevity under starvation compared to males. Combined with an even more female‐biased sex ratio upon emergence compared to adults collected from infested honey bee colonies in the field (Papach, Gonthier, Williams, & Neumann, 2019), this supports that adult female SHB have a reduced lifespan possibly due to costs associated with oviposition (Neumann et al., 2016). In general, the data are well in line with earlier reports that female insects are better adapted for prolonged periods of food scarcity than males (e.g., flies, *Drosophila leontia*, Aggarwal, 2014). Moreover, a superior starvation resistance of females seems adaptive due to the widespread ability of females to store sperm in an organ called the spermatheca (Klowden, 2003). Indeed, also female SHB are known to possess a spermatheca (Conklin, 2012) and can therefore pursue the “sit‐and‐wait” strategy after mating in infested host colonies (Neumann et al., 2016) regardless of males being present or not.

It is obvious that will be differences in food consumption prior to field collection of SHB. This is especially true if there are likely to be consistent differences in food availability given the different behavior of African versus European‐derived honey bee hosts (Neumann & Elzen, 2004). Given that food availability should be higher in the invasive range, one would therefore expect that adult SHB collected in the field from European‐derived host colonies in North America should outlive those collected from African ones because they should have had more opportunities to accumulate metabolic reserves. However, the opposite holds true. SHB that were collected in the field and reared in the laboratory in the native range in Africa lived significantly longer compared to the ones collected in the field and reared in the laboratory in the invasive range in the North America. This suggests that SHBs are better adapted to starvation in their native range compared to their new invasive range. Moreover, no differences in starvation resistance were observed between laboratory‐reared and field‐collected adult beetles in the invasive range in North America, suggesting that adult SHBs have not been under selection to withstand longer periods without food. It may well be that founder SHBs from Africa had low starvation resistance. However, this seems less likely since those founder SHBs did make it at least twice to the new range in North America in the first place, most probably with long‐range beeswax shipments from Tanzania (Ouessou Idrissou et al., 2019). This implies that they had a sufficiently high starvation resistance to at least reach the new destination and find appropriate food sources. It therefore appears as if changed selection scenarios in the invasive range, due to more predictable and constant food access, did not favor starvation resistance. Indeed, African honey bees seem to exhibit more efficient defensive behavior toward the beetles. For example, they can limit SHB access to food (Elzen et al., 2001; Schmolke, 1974). In addition, distances between nests in Africa are much greater than in North America because the vast majority of colonies are sparsely distributed wild nests compared to densely packed hives in apiaries. Furthermore, African honey bee colonies are more mobile compared to their European‐derived US counterparts (i.e., seasonal migration and absconding, Hepburn & Radloff, 1998; Spiewok et al., 2008). Therefore, adult SHBs emerging from soil may have a higher chance to not encounter a host colony in close vicinity resulting in a prolonged time of food scarcity. Moreover, long‐range migratory beekeeping, which is common in the United States, has been shown to assist the spread of SHB (Neumann & Elzen, 2004), thereby probably minimizing selection for dispersal performance (Neumann et al., 2012; Spiewok et al., 2008) compared to those in the native range.

Clearly, we cannot exclude differences in starvation resistance within the vast native range of SHB in Africa (Neumann et al., 2016). Since SHBs in North America most probably originated from Tanzania (Ouessou Idrissou et al., 2019) and the experimental SHBs were from South Africa, this could also explain the observed differences. However, those beetles from Tanzania must have survived the journey to establish a new population in 1996 in South Carolina, USA (Neumann & Elzen, 2004). Beeswax trading from Africa is the most likely invasion pathway for SHB (Ouessou Idrissou et al., 2019) and almost exclusively occurs to the United States via container ships (U.S. Trade numbers, 2020). Since it will take between 24 and 51 days for a container ship from Dar es salaam (Tanzania) to reach the port city of Charleston, South Carolina (USA) (Sea‐Distances.org), the documented starvation resistance of the SHB in North America is unlikely to be sufficient. In sharp contrast, the documented ability of field‐collected female SHB from Africa to survive up to two months without any food seems to be more than enough to reach North America via container ships. In addition, the reduced starvation resistance of the North American beetles seems to be maladaptive under African conditions (see above). In any case, given that SHBs from Tanzania actually display a reduced starvation resistance compare to the ones from South Africa, this then appears to constitute a pre‐adaptation to foster invasion success. In conclusion, we can obviously not exclude that our results reflect differences within the endemic range rather than adaptations in the novel ranges, but this seems to be unlikely.

Invasive species may adopt to novel environments, thereby promoting their success (Colautti & Barrett, 2013; Colautti & Lau, 2015). One of these cases might be the reduced starvation resistance of SHB in the invasive range in North America. Enhanced starvation resistance requires fundamental physiological changes, which are likely to result in a trade‐off with fitness (Rion & Kawecki, 2007). When resources are limited, an organism has to invest accordingly in survival, thereby often compromising it is reproduction (Kirkwood et al., 1997). Indeed, there is ample evidence suggesting a trade‐off between fecundity and longevity, energy reserves and starvation resistance (Grandison, Piper, & Partridge, 2009; Holliday, 1989; Leroi, Kim, & Rose, 1994; Partridge, Piper, & Mair, 2005). Interestingly, it has been noted that SHB can exhibit two distinct types of reproduction in association with honey bee host colonies: cryptic low‐level reproduction, with few larvae present that do not harm colonies (Ouessou Idrissou, Straub, & Neumann, 2018; Spiewok & Neumann, 2006), and overt mass reproduction, with thousands of larvae often resulting in the full structural collapse of the entire colony in a short time (Hepburn & Radloff, 1998; Neumann, Hoffmann, Duncan, & Spooner‐Hart, 2010; Spiewok et al., 2008). In the native range in Africa, mass reproduction of SHBs in association with local honey bee colonies is extremely rare (Neumann, 2017); low‐level reproduction alone seems to be sufficient to explain local SHB population size (Ouessou Idrissou et al., 2018). In sharp contrast, mass reproduction is more common in the invasive range of SHBs in the USA (Elzen et al., 1999; Neumann et al., 2016; Spiewok et al., 2008). Irrespective of which factors actually govern the higher susceptibility of colonies of European‐derived honey bees to SHBs in the invasive ranges (Neumann et al., 2016), a higher reproductive capacity (i.e., many eggs laid in a short time window) might be favored by natural selection. Thus, it seems as if a trade‐off scenario between reproduction and starvation resistance may explain the striking differences between endemic and invasive populations of SHB. It must be noted, however, that there are many other factors which may explain the invasion success of any given species (Nentwig, 2008). Therefore, the results of this study should not be generalized. Indeed, for SHB, quantitative differences in behavior between European and African honey bee subspecies, enemy release, as well as novel alternative hosts (i.e., bumblebees in invasive ranges) may all contribute to SHB invasion success (Neumann & Elzen, 2004; Neumann et al., 2016, 2018).

In conclusion, our data support the adaptive potential of invasive species (Colautti & Barrett, 2013; Colautti & Lau, 2015) due to striking differences in starvation resistance between endemic and novel ranges of SHB. The results also clearly show that SHB can live up to two months without any food. This constitutes another factor which may have contributed to SHB invasions across the globe. Assuming food availability is usually high in the invasive ranges, and trade‐offs between starvation resistance and fecundity/reproduction are common, it seems as if selection for starvation resistance during transport could set up potential trade‐offs that enhance reproduction after invasion. It would be interesting to see if this is a possible general pattern for invasive insect species.

## CONFLICTS OF INTEREST

The authors have no competing interests to declare.

## AUTHOR CONTRIBUTION

**Anna Papach:** Formal analysis (equal); Investigation (equal); Methodology (equal); Writing‐original draft (equal). **Geoffrey R. Williams:** Resources (equal); Writing‐review & editing (equal). **Peter Neumann:** Conceptualization (equal); Formal analysis (equal); Methodology (equal); Resources (equal); Supervision (equal); Writing‐original draft (equal).

## Data Availability

The complete raw data will be found at the Dryad repository. See (https://doi.org/10.5061/dryad.7sqv9s4qg).
